# Radiomics and Texture Analysis in Laryngeal Cancer. Looking for New Frontiers in Precision Medicine through Imaging Analysis

**DOI:** 10.3390/cancers11101409

**Published:** 2019-09-20

**Authors:** Carlos Miguel Chiesa-Estomba, Oier Echaniz, Ekhiñe Larruscain, Jose Angel Gonzalez-Garcia, Jon Alexander Sistiaga-Suarez, Manuel Graña

**Affiliations:** 1 Otorhinolaryngology-Head & Neck Surgery Department, Hospital Universitario Donostia, 20014 San Sebastian-Donostia, Spain; ekhinel@gmail.com (E.L.); tirititet@gmail.com (J.A.G.-G.); jasistiaga@gmail.com (J.A.S.-S.); 2 Biodonostia Health Research Institute, 20014 San Sebastian-Donostia, Spain; 3 Head & Neck Study Group of Young-Otolaryngologists of the International Federations of Oto-rhino-laryngological Societies (YO-IFOS), 13005 Marseille, France; 4 Computational Intelligence Group, Facultad de Informatica UPV/EHU, 20018 San Sebastian-Donostia, Spain; oier.etxaniz@gmail.com (O.E.); ccpgrrom@gmail.com (M.G.)

**Keywords:** larynx, cancer, radiomics, texture analysis

## Abstract

Radiomics and texture analysis represent a new option in our biomarkers arsenal. These techniques extract a large number of quantitative features, analyzing their properties to incorporate them in clinical decision-making. Laryngeal cancer represents one of the most frequent cancers in the head and neck area. We hypothesized that radiomics features can be included as a laryngeal cancer precision medicine tool, as it is able to non-invasively characterize the overall tumor accounting for heterogeneity, being a prognostic and/or predictive biomarker derived from routine, standard of care, imaging data, and providing support during the follow up of the patient, in some cases avoiding the need for biopsies. The larynx represents a unique diagnostic and therapeutic challenge for clinicians due to its complex tridimensional anatomical structure. Its complex regional and functional anatomy makes it necessary to enhance our diagnostic tools in order to improve decision-making protocols, aimed at better survival and functional results. For this reason, this technique can be an option for monitoring the evolution of the disease, especially in surgical and non-surgical organ preservation treatments. This concise review article will explain basic concepts about radiomics and discuss recent progress and results related to laryngeal cancer.

## 1. Introduction

Head and neck cancer represents the sixth most common malignancy worldwide, with around 800,000 new cases and 320,000 deaths in 2015 [1]. Laryngeal squamous cell carcinoma (LSCC) represents between 30–50% of all neoplasms in the head and neck, with 157,000 new cases diagnosed worldwide in 2012 [2]. These tumors mean a unique set of diagnostic and therapeutic challenges. The complex regional anatomy, the presence of critical structures surrounding this area, the variable appearance of primary and recurrent tumors, the significant anatomic changes related to tumor response, and high intratumoral heterogeneity make the treatment of these patients quite difficult.

Contrast-enhanced computed tomography (CT), magnetic resonance imaging (MRI), and positron emission tomography (PET) imaging are routinely acquired during the diagnosis and staging process of head and neck cancer patients. The immense data volume gathered from multiple imaging modalities in existing clinical datasets can greatly facilitate exploratory radiomic analysis. The heterogeneous composition of head and neck cancers can also be captured in a non-invasive procedure, which can serve as an essential adjunct to clinical decision-making.

The suffix “omics” characterizes different sources of large data volumes whereby this suffix is preceded by the defining name of the original materials of data collection. This leads, for example, to the description of the “genomics” in the context of genetic expression analysis, and protein analyses become “proteomics” or “metabolomics” when the metabolome is analyzed. In analogy, the newly defined “radiomics” field finds its way into clinical research where a large amount of data from radiological imaging become available for analysis [3].

Radiomics is an overall image analysis approach, the goal of which is to extract large amounts of quantitative information from radiological medical images using a variety of computational methods. Extracted image features include measurements of intensity, shape, and texture [4]. Specifically, texture analysis represents a set of tools to improve characterization of tumor heterogeneity consisting of extracting texture indices from different imaging modalities as CT, MRI, or fluorodeoxyglucose (FDG) PET/CT [5]. These imaging modalities allow clinicians to extract a large amount of quantitative features whose subsequent analysis and selection can be incorporated in clinical decision-making. These biomarkers complement, facilitate, and accelerate the advancement towards cancer precision medicine. Radiomics techniques are also able to non-invasively characterize the overall tumor accounting for heterogeneity, and quantitative imaging features can work as a prognostic or predictive biomarker, which can be derived from routine standard-of-care imaging data, providing clinicians with a fast, low-cost, and repeatable instrument for longitudinal monitoring [6,7]. 

Several studies have assessed radiomic features for different cancer types extracted from different imaging modalities [8,9,10,11,12,13]. Additionally, some studies have investigated the reproducibility and variability of radiomic features across different clinical settings [8,14,15,16], while others have reported the significant predictive and prognostic power of radiomic features. The relation between radiomic features and tumor histology [17,18,19], tumor grades or stages [18], patient survival [8,9,20,21,22], metabolism [23], and various other clinical outcomes have also been reported [9,18,24,25]. Furthermore, some radio-genomic studies have reported associations between radiomic features and underlying gene expression patterns [8,11,13,26,27]. These reports indicate that radiomics could improve individualized treatment selection and monitoring. 

Radiomics represents a non-invasive and relatively cost-effective technique [8,28] that can expand the scope of medical imaging in clinical oncology, and its application can provide important day-to-day information regarding rapid anatomic change and tumor response during the course of treatment. However, several key components are necessary to transition radiomics and texture analysis in larynx cancers from exploratory studies to large-scale implementation as a clinical toolset. This is the reason why in this review the authors try to summarize and expose the most relevant concepts and challenges of the technique and its application in laryngeal cancer. 

### 1.1. Radiomics and Texture Analysis Software Available

The most common image features are those based simply on voxel intensity values within a region of interest (ROI). Similar but unique features may also be extracted from histograms of intensity values and Gaussian functions fitted to these histograms. However, other spatial features may be calculated from the shape of the ROI. Texture features in the head and neck are based on the same matrices that are utilized in other sites. Examples of these include the gray-level co-occurrence matrix (GLCM), the gray-level run length matrix (GLRLM), the neighborhood intensity difference matrix (NIDM), neighborhood gray-level dependence matrix (NGLDM), and the intensity size-zone matrix (ISZM) [29,30,31,32,33,34]. Other feature extraction methods are based on filters such as Fourier transform, Gabor transform, Laplacian of Gaussian filter (LoG), and multi-scale wavelet decompositions [35,36,37,38]. After processing the ROI according to the parent matrix or filter method, statistical features such as coarseness, business, correlation, entropy, and energy are calculated. 

There are multiple open-source, in-house developed, and commercial software solutions that facilitate the exploration and development of radiomics in head and neck cancer. 

#### 1.1.1. Open-Source Software

- IBEX (Imaging Biomarker Explorer): developed by Zhang et al., described as an “open infrastructure software platform that flexibly supports common radiomics workflow tasks such as multimodality image data import and review, development of feature extraction algorithms, model validation, and consistent data sharing among multiple institutions.” [39]. IBEX is compatible with CT, PET, and MRI modalities.
- MazDa is another open-source solution for texture analysis that has been validated through multi-institutional studies [40]. This software is built primarily for MRI texture analysis and supports various feature selection methods for model generation.
- Chang-Gung Image Texture Analysis (CGITA) is yet another open-source texture analysis tool, built in the MATLAB environment. The software supports numerous heterogeneity indices, user-defined calculations, and batch processing with a focus on molecular imaging. CGITA supports CT, PET, and MRI images [41].

#### 1.1.2. In-House Development of Radiomic Analysis

Beyond open-source software tools, a number of groups have also developed in-house tools for radiomic analyses, often in the MATLAB environment; however, they are not publicly available to our knowledge [14,42,43,44,45], thus cannot be validated. One such example is a modified version of Computational Environment for Radiotherapy Research (CERR) used for texture analysis.

#### 1.1.3. Commercial Solutions Software for Radiomic Analysis

- TexRAD is a commercial software that uses a LoG special filter to delineate fine, intermediate, and coarse textures in a ROI for subsequent analysis. This software contains various decision support tools for thoracic and gastrointestinal imaging and has also demonstrated applicability in head and neck cancer textural analysis [46].

### 1.2. Radiomics Workflow

**Figure 1:**
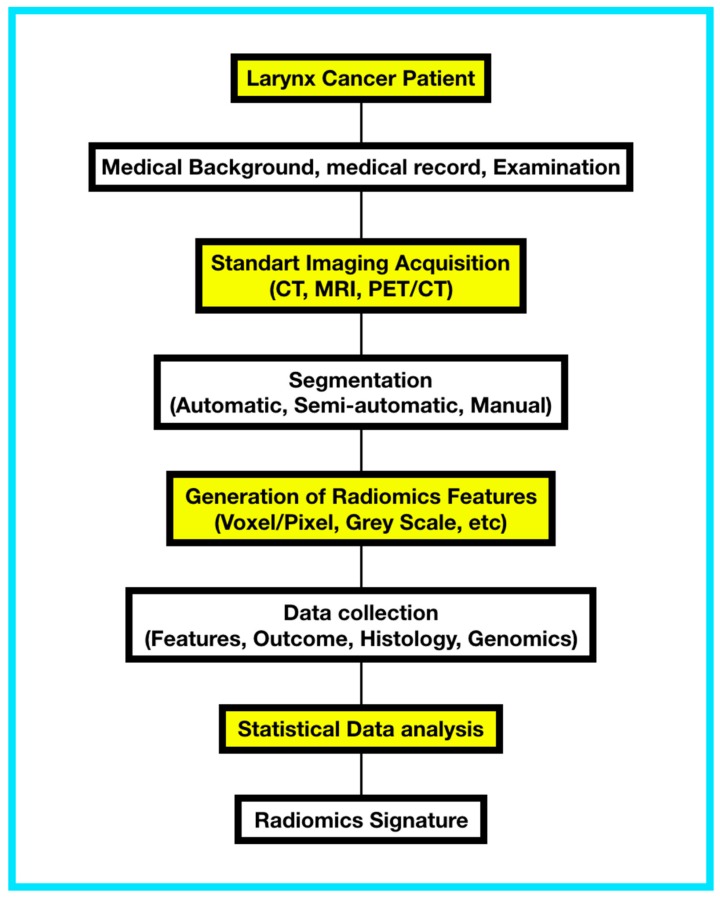
Workflow for creation of a radiomic signature in a larynx cancer patient.

- High-quality, standardized imaging data must be acquired. The region of interest (ROI), represented by the tumor, metastasis, or parts of it is manually/automatically identified, and the volume of interest (VOI) is defined.
- Collections of datasets from clinical practice can be gathered to perform retrospective analysis in order to obtain the basic radiomic feature extraction and statistical and predictive systems for prospective analysis.
- Definition and segmentation of the ROI: In each subject, capture of radiomic imaging data can be performed using a manual, semiautomatic or automatic approach.
- Radiomic feature extraction: These features are extracted from the tumor ROI concerning information about image shape, intensity, and texture. Features can be constructed by statistical means, such as co-occurrence matrices, or by selecting the coefficients of image transformations, such as wavelet-based image decomposition and analysis.
- Multi-source fusion data analysis: Defining associations between radiomic features and clinical data, outcome, treatment responses, histopathological data. Mixed analysis, e.g., including gene expression (“radiogenomics”) is achieved through data fusion schemes like canonical correlation analysis (CCA).
- Machine learning algorithm application: Predictive/discriminant functions can be trained and validated over the radiomic feature collected from retrospective data in order to be refined and applied in prospective studies. Model building procedures include logistic regression, support vector machines (SVM), random forests (RF), and artificial neural networks including deep learning approaches. Radiomics can be fused with survival analysis for prognostic studies. [8,47,48,49]. (Figure 1)

## 2. Radiomics and Laryngeal Cancer

In the last few decades, treatment approaches for larynx squamous cell carcinoma have shifted toward organ-preserving strategies, with the aim of limiting functional impairments associated with total laryngectomy and improving patient’s quality of life [50]. While organ-preserving trials have provided strong evidence that well-selected patients may benefit from organ-preservation strategies, there remains substantial controversy on the optimal management of these patients [51]. In fact, despite improvement in radiotherapy techniques and systemic treatments, relapse rates in locally advanced laryngeal cancer after organ-preserving treatment remain high, with rates of loco-regional recurrence at 5 years reaching 30–40% [52,53,54]. In addition, observational data suggest that five-year survival rates in laryngeal cancer have decreased [55].

The larynx anatomy represents one of the most complex localizations in the head and neck region, due to the presence of important structures surrounding the larynx. Radiomics can provide a more comprehensive characterization of entire tumors, and hence are likely to capture the intra-tumor heterogeneity. This is the reason why a better tumor characterization through the use of imaging biomarkers has the potential to provide insightful information for outcome prediction, treatment selection, and disease progression in those patients. Therefore, it can be considered as a crucial factor for precision oncology and related research [56,57,58].

Radiomics, with special emphasis in image texture analysis, has demonstrated exciting promise in several distinct areas in larynx cancer research. Some of them are summarized next.

- Tumor segmentation and pathologic classification in surgical and non-surgical patients.
- Anatomical extension: Paraglottic space, thyroid cartilage, cryco-aritenoid joint, cryco-thyroid membrane.
- Risk stratification.
- Prognostic or predictive biomarker.
- Monitorization of alterations in normal tissue as a sequelae of radiotherapy dose deposition.

We have identified eight studies including patients with larynx cancer in the indexed literature search (Table 1). Guezennec, C. et al. evaluated the prognostic value of texture indices related to overall survival (OS). In this study 32/284 (11%) patients with larynx cancer were included. The imaging acquisition method was the FDG PET/CT, and the discriminant features were metabolic tumor volume, correlation, entropy, energy, and coarseness [5]. Ulrich, E. et al. evaluated the utility of radiomic feature analysis from 18F-fluorothymidine positron emission tomography (FLT PET) obtained at baseline in prediction of treatment response in patients with head and neck cancer. In this study 2/30 (6.7%) with larynx cancer were included. The authors considered nine discriminant features. Their results suggest that the most homogenous lesions at baseline were associated with better prognosis [4].

From the group of Bogowicz, M. et al. we identified three papers. In one of them the authors tried to show the association of post-radiochemotherapy (RCT) PET radiomics with local tumor control. To this effect, the authors evaluated the models against two radiomics software implementations, 11 out 128 (9%) patients with larynx cancer were included, and the study showed the potential of post-RCT FDG-PET radiomics for early identification of patients with a high risk of local tumor recurrence [57]. In another study, the authors tried to predict local tumor control after RCT of head and neck squamous cell carcinoma and Human Papilloma Virus (HPV) status using CT radiomics. A population of patients and healthy controls were included. A radiomic signature, comprising three features, was significantly associated with local control showing that tumors with the most heterogeneous CT density distribution are at risk for decreased local control [58]. In a third study, the authors compared the use of PET and CT radiomics for prediction of local tumor control in head and neck squamous cell carcinoma. Eight percent of patients with larynx cancer were included and 569 radiomic features were extracted from both contrast-enhanced CT and 18F-FDG PET. The authors concluded that the most homogenous tumors in CT density with a focused region of high FDG uptake indicated better prognosis. However, the CT radiomics-based model overestimated the probability of tumor control in the poor prognostic group [59].

Ou, D. et al. developed a radiomics signature model to estimate overall survival in patients with locally advanced head and neck squamous cell carcinomas treated with concurrent chemoradiotherapy or bioradiotherapy to assess its incremental value to HPV and clinical risk factors for individual OS estimation and also to explore its predictive value. The imaging acquisition method used was the CT. A total of 544 radiomics image features were defined and were divided in four groups: (I) tumor intensity, (II) shape, (III) texture, and (IV) wavelet features, but it was found that only 24-feature-based signature significantly predicted for OS and progression-free survival (PFS). Patients with larynx cancer were included in the study, but the number of patients was not described [60]. Zhang, H. et al. examined the association between overall survival and the baseline CT imaging measurements and clinical variables. The study includes 21/72 (29%) with a locally advanced larynx cancer. The imaging acquisition was performed using a CT, which demonstrated that primary mass entropy and skewness measurements were associated to OS. After the multivariate Cox regression analysis incorporating clinical and imaging variables, results indicated that primary mass size, N stage, primary mass entropy, and skewness measurements were independently associated to OS [46]. Finally, Kuno, H. et al. tried to assess the utility of texture analysis for the prediction of treatment failure in primary in head & neck squamous cell carcinoma (HNSCC) treated with RCT. In this study 19/62 patients (31%) with larynx cancer were included. The imaging acquisition method was the CT, and the multivariate analysis revealed that three histogram features (geometric mean, harmonic mean, and fourth moment) and four gray-level run-length features (short-run emphasis, gray-level nonuniformity, run-length nonuniformity, and short-run low gray-level emphasis) were significant predictors of outcome after adjusting for clinical variables [61].

However, it is necessary to be cautious when interpreting or comparing these results. Studies included all kind of head and neck tumors making it difficult to perform a proper meta-analysis or to extract any type of conclusion or propose any statement. Larynx cancer has a different behavior compared with other head and neck tumors; for this reason, new studies related exclusively to larynx cancer must include radiomics features as a valid biomarker in the diagnosis and follow-up of larynx cancer patients. Moreover, software available should be easily accessible, measurable, and reproducible, so their stability with regard to the measurements should be verified. To do this, the evaluation should be performed in a representative, possibly large and standardized cohort where well-defined parameters are sufficiently assessed. After definition of the biomarker signature, it should also be reevaluated in a second cohort, preferably independent in another institution. Furthermore, a prospective validation is necessary in order to confirm the reliability of this technique.

## 3. Precision Medicine, Big Data, and Machine Learning in Larynx Cancer 

**Figure 2:**
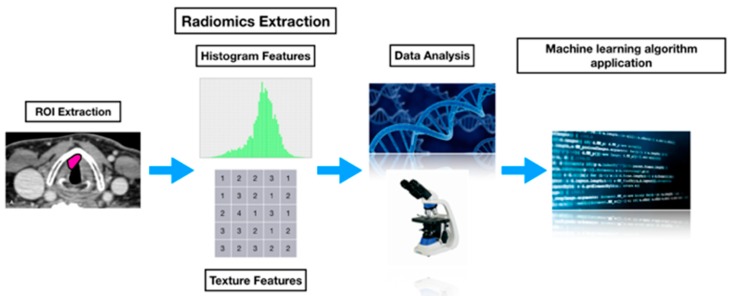
Radiomics analysis workflow. Image segmentation is performed on computed tomography (CT) images. Experienced radiologists delineate the volume of interest covering the whole tumor by stacking up the region of interest slice by slice. Radiomics features are extracted including shape- and size-based features, first-order histogram features, and textural features. The data are analyzed, and clinical application is tested.

The aim of precision medicine is to accurately define diseases in order to find personalized and individual therapies. This approach should improve healing, reducing the spectrum of side effects [3]. Large data volumes are acquired during the disease evolution, thus providing various options for the definition of appropriate biomarkers or marker patterns in different stages of the disease, on different steps of the analysis, and of most diverse materials and data sources. To exploit this big amount of retrospective data, using mathematical algorithms, a quantitative high-throughput extraction of radiological features based on meta-datasets (DICOM format) should be carried out to evaluate all those image features that cannot be perceived with the human eye against the clinical outcome as the final objective reference for predictive analysis, thus creating a place for radiomics and texture analysis in the spectrum of precision medicine [62] (Figure 2).

The development of “big data” and “machine learning” techniques have increased our possibilities to identify the different characteristics of a disease containing information about prognosis and diagnosis with regard to the status, the outcome, and the therapeutic response. Those variables can be analyzed in the context of clinical data and the course of the disease while the clinical data still serve as diagnostic and prognostic parameters, helping clinicians to improve decision-making protocols looking for precision medicine.

According to the definition proposed in “Big data in health research: An EU action plan”, “Big data in health” encompasses high volume, high diversity, biological, clinical, environmental, and lifestyle information collected from single individuals to large cohorts, in relation to their health and wellness status, at one or several time points [63]. However, big data come from a variety of heterogeneous information sources, such as clinical trials, electronic health records, patient registries, or clinical databases, posing big problems for interoperability and fusion of the information towards building decision support systems (DSS) with clinical value.

Machine learning can be broadly defined as the collection of computational methods using data to improve performance or make accurate prediction. These programmable methods can learn from the data, and hence automate and improve the prediction process. Recently Parmar et al. assessed a large panel of machine-learning methods for overall survival prediction of head and neck cancer patients. They investigated 12 machine-learning classifiers belonging to the 12 classifier families: Bagging (BAG), Bayesian (BY), boosting (BST), decision trees (DT), discriminant analysis (DA), generalized linear models (GLM), multiple adaptive regression splines (MARS), nearest neighbors (NN), neural networks (Nnet), partial least square and principle component regression (PLSR), random forests (RF), and support vector machines (SVM). In this study the authors demonstrated the also showed high prognostic performance and stability of machine learning methods applied to Radiomics features [64].

In head and neck cancer and larynx cancer field, the routine non-contrast-enhanced CT performed for radiotherapy planning can constitute nowadays the basis for radiomics development. Examples are especially aimed at providing a prognostic biomarker in different head and neck cancers [18,64,65]. MRI can also provide a higher number of features suitable for diagnostic and prognostic purposes, especially with functional sequences, such as diffusion-weighted imaging (DWI) and apparent diffusion coefficients (ADC). The large amount of data generated can be used to elaborate an algorithm for automatic diagnosis of laryngeal cancer and to predict outcome based on pre- and post-RCT treatment, using those CT or MRI during the follow-up.

At this moment, machine learning or big data are not used commonly in head and neck or larynx cancer research. This can be explained by the limited numbers of patients still recruited mainly for traditional clinical studies. However, some specific machine learning and big data techniques are finding an increasing application in radiomics [66].

## 4. Radiomics Limitations

Nowadays, variations in radiomics workflow among different researchers lead to obtaining different results, compromising the reproducibility of the technique. Moreover, this makes it more difficult to establish a validated and useful radiomic signature.

### 4.1. Imaging Acquisition

The type of machine used during the image acquisition, the different slice thickness, reconstruction matrices, configurations, fields of view, the time point of the administration and type of contrast as well as the time point of the investigation itself during the course of illness may lead to relevant differences in the image. All these parameters have an important correlation with the VOI sizes and the ROI definition during segmentation affecting radiomic markers [7].

### 4.2. Image Segmentation

Image segmentation represents one of the most important steps during radiomic workflow. It can be performed manually, semiautomatically or automatically. Manual segmentation represents a time-consuming procedure. This also needs to be carried out by a specialist, and a high interobserver variability can be expected. Automatic segmentation represents a more reproducible and faster way to do our segmentation and is very useful for large imaging datasets. Semiautomatic segmentation needs an interaction between the observer and software. Most often, the observer needs to define the VOI and may refine the semiautomatic segmentation results manually. Although some automatic and semiautomatic segmentation methods exist, they are not suitable for every VOI and thus need to be upgraded for certain problems and used with standardized adjustments when they are applied [7].

### 4.3. Feature Extraction

Imaging features are extracted from the VOI areas defined by the segmentation. Every feature represents one part from the whole radiomic concept. The tumor intensity is quantified by an intensity histogram that displays the three-dimensional fractional volume data for the range of voxel values. Shape data contain values like the total volume, surface area, compactness, and actual form of the lesion. Texture-based features are compiled by mathematical algorithms that deliver second-order statistics or co-occurrent matrix features. Hundreds of values may be generated containing information about additional qualities like densities, homogeneity, grey shades, clusters, correlations between those in different settings, and much more.

A common challenge in radiomics is to define a non-redundant set of imaging biomarkers from the vast number of extracted features, and to improve the radiomic performance, redundancies should be excluded [7]. To do this, after feature extraction, the use of some statistical methods or supporting our workflow with machine learning allows us to select the most precise features reducing the spectrum to those validated.

### 4.4. Image Processing

To make a successful image processing procedure, an appropriate bioinformatics approach must be chosen that is able to cope with big data, to obtain a reproducible and solid biomarker signature. However, this make it necessary to perform careful data management to improve the study results and diminish the influence of outliers, blurring values, interobserver variability, and diffuse readings [8]. Hundreds of radiomic features can be extracted from each tumor; as many patients as possible should ideally be integrated into the study. This is of course often limited—even in multicenter studies. Commonly, those relevant markers are determined in retrospective cohort studies. When a radiomic signature has been identified, it should be verified in an independent cohort in a prospective study [6].

## 5. Future Direction

Nowadays, radiomic features can predict some tumor characteristics linked to survival in some head and neck cancer [67]. Moreover, some data from the study of Aerts et al. [9] in non-small cell lung cancer suggest that when the TNM classification is combined with radiomics signature, the performance is significantly better than TNM alone, suggesting complementary information for prognosis. However, as the larynx is one of the most complex anatomical and functional organs in the head and neck region, more studies are necessary to determine how radiomic signatures can improve phenotype analysis and prognosis prediction in larynx cancer due to its different behavior in comparison with other head and neck cancers.

## 6. Conclusions

The metadata summarized in this paper suggest that there is great potential for radiomics and texture analysis techniques for improving upon multiple aspects of the tumor assessment, risk stratification, and outcome evaluation aspects in laryngeal cancer therapy. An effort towards standardization of radiomics algorithms and specific acquisition parameters is necessary for the oncologic community to define the role of radiomics and texture analysis techniques in a manner that the clinical practice takes in. New studies about specific head and neck sublocalization are needed, including the larynx.

## Figures and Tables

**Table 1:** Studies about radiomics and textural analysis including larynx cancer patients.

Ref.	Number of Patients	Image Acquisition	Treatment	Significant Features	Study Objective
[5]	32	Fluorodeoxyglucose (FDG) positron emission tomography (PET)/computed tomography (CT)	Surgery, radiochemotherapy (RCT), Radiotherapy (RT), palliative (not specified by localization)	Metabolic tumor volume, correlation, entropy, energy, and coarseness.	Prognostic value of texture indices over overall survival (OS).
[4]	2	F-fluorothymidine positron emission tomography (FLT PET)	RCT	Nine features were considered significant. Their results suggested that homogenous lesions at baseline were associated with better prognosis.	Evaluate the utility of radiomic feature analysis from FLT PET obtained at baseline in prediction of treatment response in patients with head and neck cancer.
[57]	11	FDG PET/CT	RCT	80 PET radiomic features yielded intraclass correlation coefficient >0.8 in the comparison between the implementations. The change of implementation caused high variability of concordance index (CI) in the univariable analysis. However, both final multivariable models performed equally well in the training and validation cohorts (CI > 0.7) independent of radiomics implementation.	Association of post (RCT) PET radiomics with local tumor control.
[60]	Not specified	CT	RCT/Bio-Radiotherapy (BRT)	544 radiomics image features were defined and were divided in four groups: (I) tumor intensity, (II) shape, (III) texture, and (IV) wavelet features.	Develop a radiomics signature to estimate OS in patients with locally advanced head & neck squamous cell carcinoma (HNSCC) treated with concurrent RCT or BRT and assess its incremental value to Human Papilloma Virus (HPV) and clinical risk factors for individual OS estimation and also to explore its predictive value.
[46]	21	CT	Cisplatin, 5-fluorouracil, and docetaxel (TPF) Induction Chemotherapy (ICT)	Primary mass entropy and skewness measurements with multiple spatial filters were associated with OS. Multivariate Cox regression analysis incorporating clinical and imaging variables indicated that primary mass size, N stage, primary mass entropy and skewness measurements with the 1.0 spatial filter were independently associated with OS.	Examine the association between overall survival and the baseline CT imaging measurements and clinical variables.
[61]	19	CT	Not specified	Multivariate analysis revealed that three histogram features (geometric mean, harmonic mean, and fourth moment) and four gray-level run-length features, (short-run emphasis, gray-level nonuniformity, run-length nonuniformity, and short-run low gray-level emphasis) were significant predictors of outcome after adjusting for clinical variables.	Assess the utility of texture analysis for the prediction of treatment failure in primary HNSCC treated with RCT.
[58]	4	CT	RCT	A radiomic signature, comprising three features, was significantly associated with local control showing that tumors with the most heterogeneous CT density distribution are at risk for decreased local control.	This study aimed to predict local tumor control (LC) after RCT of HNSCC and HPV status using CT radiomics.
[59]	10	CT/18F-FDGPET/CT	RCT	569 radiomic features were extracted from both contrast-enhanced CT and 18F-FDG PET. The most homogenous tumors in CT density with a focused region of high FDG uptake indicated better prognosis. However, the CT radiomics-based model overestimated the probability of tumor control in the poor prognostic group.	Comparison of PET and CT radiomics for prediction of local tumor control in HNSCC.
